# Supplementary material for: Comparative genomics provides new insights into the diversity, physiology, and sexuality of the only industrially exploited tremellomycete: Phaffia rhodozyma
Source: BMC Genomics. 2016 Nov 9;17:901. doi: 10.1186/s12864-016-3244-7 (PMC5103461; doi:10.1186/s12864-016-3244-7)
Supplement: Additional file 6: — List of orphan genes with links to PFAM (related to Additional file 1: Table S1). (ZIP 1428 kb) [file 12864_2016_3244_MOESM6_ESM.zip › BLAST_HTML_FTR/G02048_P.html]

BLAST Search Results


```
BLASTP 2.2.27+


Reference:
Stephen F. Altschul, Thomas L. Madden, Alejandro A. Schäffer,
Jinghui Zhang, Zheng Zhang, Webb Miller, and David J. Lipman (1997),
"Gapped BLAST and PSI-BLAST: a new generation of protein database
search programs", Nucleic Acids Res. 25:3389-3402.


Reference for
composition-based statistics:
Alejandro A. Schäffer, L. Aravind, Thomas L. Madden, Sergei
Shavirin, John L. Spouge, Yuri I. Wolf, Eugene V. Koonin, and
Stephen F. Altschul (2001), "Improving the accuracy of PSI-BLAST
protein database searches with composition-based statistics and
other refinements", Nucleic Acids Res. 29:2994-3005.


Database: nr
           71,551,133 sequences; 26,053,659,533 total letters


Query= G02048_P

Length=117
                                                                      Score     E
Sequences producing significant alignments:                          (Bits)  Value

emb|CED83191.1|  hypothetical protein [Xanthophyllomyces dendrorh...   230    4e-75


 >emb|CED83191.1| hypothetical protein [Xanthophyllomyces dendrorhous]
Length=116

 Score =  230 bits (586),  Expect = 4e-75, Method: Compositional matrix adjust.
 Identities = 115/116 (99%), Positives = 116/116 (100%), Gaps = 0/116 (0%)

Query  1    MSLRTFSRSLFASLGRVPAIEAAASRSYASKPPAKQNVSASTADRTDSNQAGPYQPADYT  60
            MSLRTFSRSLFASLGRVPAIEAAASRSYASKPPAKQNVSASTADRTDSNQAGPYQPADYT
Sbjct  1    MSLRTFSRSLFASLGRVPAIEAAASRSYASKPPAKQNVSASTADRTDSNQAGPYQPADYT  60

Query  61   TEVAPEGLGALPGQGGKQGEMTNESGSAHVDSQARSSNETKETVEGPLANAGKSKQ  116
            TEVAPEGLGALPGQGGKQGEMTNESGSA+VDSQARSSNETKETVEGPLANAGKSKQ
Sbjct  61   TEVAPEGLGALPGQGGKQGEMTNESGSANVDSQARSSNETKETVEGPLANAGKSKQ  116


Lambda      K        H        a         alpha
   0.307    0.122    0.332    0.792     4.96 

Gapped
Lambda      K        H        a         alpha    sigma
   0.267   0.0410    0.140     1.90     42.6     43.6 

Effective search space used: 639098023296


  Database: nr
    Posted date:  Sep 23, 2015 12:05 AM
  Number of letters in database: 26,053,659,533
  Number of sequences in database:  71,551,133


Matrix: BLOSUM62
Gap Penalties: Existence: 11, Extension: 1
Neighboring words threshold: 11
Window for multiple hits: 40
```
